# *Salmonella* Enteritidis in Broiler Chickens, United States, 2000–2005

**DOI:** 10.3201/eid1212.060653

**Published:** 2006-12

**Authors:** Sean F. Altekruse, Nathan Bauer, Amy Chanlongbutra, Robert DeSagun, Alecia Naugle, Wayne Schlosser, Robert Umholtz, Patricia White

**Affiliations:** * US Department of Agriculture Food Safety and Inspection Service, Washington, DC, USA;; † US Department of Agriculture Food Safety and Inspection Service, College Station, Texas, USA;; ‡ US Department of Agriculture Food Safety and Inspection Service, Omaha, Nebraska, USA

**Keywords:** Salmonella Enteriditis, Salmonella infection, poultry, research

## Abstract

US Department of Agriculture Food Safety and Inspection Service (FSIS) data on *Salmonella enterica* serotype Enteritidis in broiler chicken carcass rinses collected from 2000 through 2005 showed the annual number of isolates increased >4-fold and the proportion of establishments with *Salmonella* Enteritidis–positive rinses increased nearly 3-fold (test for trend, p<0.0001). The number of states with *Salmonella* Enteritidis in broiler rinses increased from 14 to 24. The predominant phage types (PT) were PT 13 and PT 8, 2 strains that a recent Foodborne Diseases Active Surveillance Network (FoodNet) case-control study associated with eating chicken. FSIS is directing more sampling resources toward plants with marginal *Salmonella* control to reduce prevalence in products including broilers. The policy targets establishments with common *Salmonella* serotypes of human illness, including *Salmonella* Enteritidis. Voluntary interventions should be implemented by industry.

## Abstract 2

TOC summary line: Greater sampling and monitoring efforts are needed to reverse a significant increase in prevalence.

During the 1990s, Salmonella enterica serotype Enteritidis briefly surpassed S. Typhimurium as the predominant Salmonella serotype isolated from humans in the United States (*1*). Eggs were frequently implicated as the cause of outbreaks of human infection (*2**,**3*), and the outbreak strain was often detected in the implicated egg production flock (*4*). After egg producers implemented quality assurance programs in the late 1990s, human Salmonella Enteritidis infection rates decreased by ≈50% (*1*).

Recently, 2 US case-control studies in Foodborne Diseases Active Surveillance Network (FoodNet) sites identified eating chicken as a risk factor for sporadic human Salmonella Enteritidis infection (*5**,**6*), replicating findings of a case-control study performed in England in the late 1980s (*7*). While the overall incidence of human salmonellosis in FoodNet sites was lower in 2005 than in the mid-1990s, the incidence of Salmonella Enteritidis infections was ≈25% higher (*8*). We present US Department of Agriculture (USDA) Food Safety and Inspection Service (FSIS) Salmonella testing program data collected from 2000 to 2005 that suggest a need for interventions to prevent the emergence of this Salmonella serotype in broiler chickens in the United States.

## Methods

### FSIS Salmonella Testing Program

As of January 2000, an FSIS performance standard for Salmonella was set for all establishments that slaughter US broiler chickens (*9*). Establishments that slaughtered >20,000 chickens per year were eligible for FSIS regulatory Salmonella testing. These establishments accounted for >95% of raw poultry marketed in the United States.

The sampling frame for the present study included all eligible FSIS-inspected establishments. Each month, eligible facilities were randomly selected for Salmonella testing to begin in the following month. In each broiler slaughter setting that was tested, 1 broiler chicken carcass rinse (hereafter referred to as broiler rinse) was collected per day for 51 days of operation. The 51 broiler rinses constitute a "Salmonella set." Sets were scheduled approximately once a year. When a plant did not meet the Salmonella performance standard, a follow-up set was scheduled. To limit bias, this report does not include data from follow-up sets.

Carcasses were collected after they exited the chiller, downstream from the slaughter line. The chiller is designed to bring carcass temperatures down to the refrigeration range. The postchill collection site was selected as the sampling site because interventions for pathogen reduction are generally located before this point.

### Broiler Rinse Collection

Carcasses were collected after they exited the chiller and aseptically placed in a sterile bag. A 400-mL volume of buffered peptone water was added to the carcass in the bag. Half the volume was poured into the interior cavity and the other half over the skin. The carcass was rinsed with a rocking motion for 1 minute at a rate of ≈35 cycles per minute. After the carcass was removed from the bag, the rinse was poured into a sterile container and shipped on a freezer pack by overnight mail to 1 of 3 FSIS laboratories (Athens, GA; Alameda, CA; St. Louis, MO, USA) for analysis (*10*).

### Microbiologic Testing

Testing of broiler rinses for Salmonella was performed by using standard FSIS isolation methods (*11*). Before October 2003, an immunoassay system (Assurance polyclonal enzyme immunoassay, BioControl Systems, Inc., Bellevue, WA, USA) was used to screen enrichment broths for Salmonella. Beginning in October 2003, Salmonella gene amplification (BAX System PCR Assay, DuPont Qualicon, Wilmington, DE, USA) was performed on lysed cells after overnight incubation in buffered peptone broth (35°C). Broiler rinses that tested positive on the screening test were cultured for Salmonella with standard methods (i.e., selective enrichment, plating, serologic and biochemical confirmation). Three presumptive Salmonella colonies with the predominant colony form were selected from each plate for biochemical and serologic confirmation. One confirmed Salmonella isolate was sent to the National Veterinary Services Laboratories (NVSL, USDA-APHIS-VS, Ames, IA, USA) for Salmonella serotyping (*12*).

Beginning in 2001, isolates of Salmonella Enteritidis were phage typed at NVSL (*13*). Because the predominant Salmonella Enteritidis phage types were clonal (*6**,**14*) and pulsed-field gel electrophoresis and antimicrobial susceptibility patterns were not available on all isolates during the study period, no further characterization of the isolates was performed for this report.

### Analysis

Analysis was restricted to Salmonella sets performed in calendar years 2000–2005. A χ^2^ test (2-sided) was used to test trends for annual percent of Salmonella Enteritidis isolates among Salmonella-positive broiler rinses and all analyzed broiler rinses, respectively. A χ^2^ test for trend was also performed to assess the percent of establishments tested annually with Salmonella Enteritidis–positive broiler rinses, with subanalyses by establishment size. Approximately two thirds of establishments were large (>500 employees), one fourth were small (<500 but >10 employees), and 5% were very small (<10 employees). In addition, a χ^2^ test for trend was performed on the number of isolates per Salmonella Enteritidis–positive establishment, by year (SAS version 9.1, SAS Institute, Inc., Cary, NC, USA).

The number of positive broiler rinses per state was plotted on a US map that showed the geographic density of broiler chicken production by county for the year 2002 (*15*). Results were plotted for 2 periods: calendar years 2000–2002 and 2003–2005. Phage types of isolates were tabulated by year.

The present study preceded a new FSIS policy to control Salmonella. The new policy emphasizes improvement in Salmonella control in product classes that have not reduced Salmonella prevalence in the past decade, such as broilers, and focuses on plants that test positive for common serotypes of human illness, such as Salmonella Enteriditis (*16*).

## Results

During the 6-year study period, 280 (0.5%) Salmonella Enteritidis isolates were recovered from 51,327 broiler rinses (Table 1). From 2000 to 2005, the proportion of Salmonella isolates that were Salmonella Enteritidis increased (test for trend, p<0.0001). The percentage of all broiler rinses that tested positive also increased (test for trend, p<0.0001).

**Table 1:** *Salmonella* Enteritidis (SE) isolates per year as a proportion of *Salmonella-*positive broiler rinses and total broiler rinses, 2000–2005

Year	No. SE isolates	Salmonella-positive rinses	SE as a proportion of all salmonellae (%)*	No. rinses tested	SE-positive rinse as a proportion of all rinses*
2000	23	914	2.5	10,057	0.2
2001	17	1,065	1.6	8,955	0.2
2002	33	1,059	3.1	9,183	0.4
2003	29	828	3.5	6,468	0.5
2004	58	957	6.1	7,072	0.8
2005	120	1,559	7.7	9,592	1.3
Total	280	6,382	4.4	51,327	0.5

*Test for trend, p<0.0001; SE isolates as a proportion of Salmonella isolates and all broiler rinses by year.

Overall, 90 establishments tested positive from 2000 through 2005. The number of establishments testing positive increased from 17 (9%) of 197 in 2000 to 47 (25%) of 187 in 2005 (test for trend, p<0.0001, Table 2). The increase in the number of positive establishments per year remained significant after stratification by large versus small establishment size. While most establishments with Salmonella Enteritidis–positive broiler rinses were large, in most years, a higher proportion of the small establishments that were tested had positive rinses.

**Table 2:** Establishments with *Salmonella* Enteritidis (SE)–positive broiler rinses, by establishment size,* 2000–2005

Year	All*		Large†		Small‡	
	No. tested	SE positive (%)	No. tested	SE positive (%)	No. tested	SE positive (%)
2000	197	17 (9)	128	9 (7)	60	8 (13)
2001	186	15 (8)	111	10 (9)	61	5 (8)
2002	185	22 (12)	123	17 (14)	53	5 (9)
2003	143	25 (17)	103	17 (17)	37	8 (22)
2004	160	25 (16)	111	16 (14)	43	9 (21)
2005	187	47 (25)	126	32 (25)	48	15 (31)

*Establishment size: large >500; small >10 to <500; very small <10 employees. (SE was not isolated in the 54 sets from very small establishments.) Ninety establishments had SE-positive broiler rinses; 63 (70%) were large and 27 (30%) were small. †Test for trend, p<0.0001. ‡Test for trend, p<0.01.

**Figure 1:**
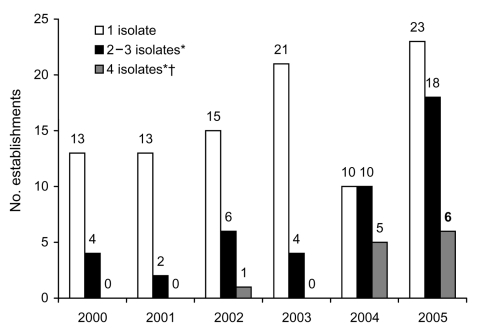
Number of Salmonella Enteritidis (SE)–positive broiler rinses, by establishment, 2000–2005. *p<0.01, test for trend. †4 establishments had 2 broiler sets in 2005; the mean number of SE isolates per set in 2005 is presented for these establishments.

During the 6-year study period, the proportion of Salmonella Enteritidis–positive establishments with multiple positive broiler rinses per year also increased significantly (p<0.01, test for trend, Figure 1). In addition, the proportion of establishments with >4 positive broiler rinses per year (of 51 broiler rinse tests per set) increased, beginning in 2002.

**Figure 2:**
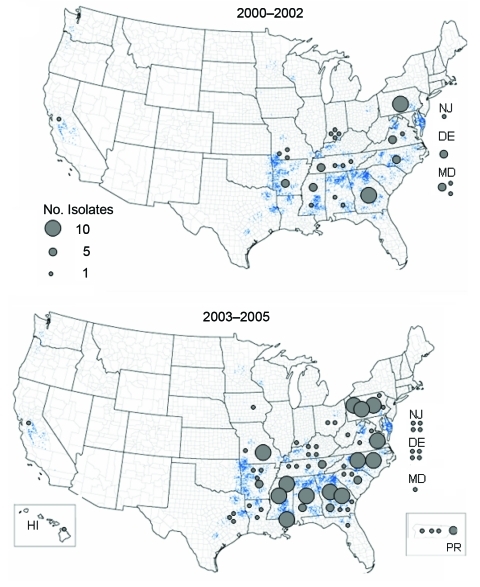
Geographic distribution of Salmonella Enteritidis isolates in broiler rinses in the first and second half of the study period (2000–2002 vs. 2003–2005). Each blue dot represents 2 million broilers produced in 2002. Broiler production data: US Department of Agriculture National Agricultural Statistics Service.

From 2000 to 2002, Salmonella Enteritidis was isolated from broiler rinses in 14 states, compared with 24 states from 2003 to 2005 (Figure 2). Phage type (PT) 13 was predominant, accounting for half of all isolates, followed by Salmonella Enteritidis PT 8, which accounted for more than one third of isolates (Table 3). In 2005, the number of isolates that were PT 8 increased >3-fold compared with 2004.

**Table 3:** *Salmonella* Enteritidis (SE) phage types from broiler rinses, 2001–2005*

Year	SE isolates no.	Phage type			
		PT 13 no. (%)	PT 8 no. (%)	Other† no. (%)	No data‡ no. (%)
2001	17	11 (65)	4 (24)	1 (6)	1 (6)
2002	33	12 (36)	8 (24)	5 (15)	8 (24)
2003	29	13 (45)	14 (48)	1 (3)	1 (3)
2004	58	37 (64)	15 (26)	4 (7)	2 (3)
2005	120	56 (47)	50 (42)	9 (8)	5 (4)
Total	257	129 (50)	91 (35)	20 (8)	17 (7)

*Phage type (PT) data were not available for 2000. Row percents do not sum to 100 because of rounding. †Other phage types: 13a (7); PT 23 (7); PT 28 (3); PT 2 (2); PT 14B (1). ‡No phage type data were available for 17 isolates.

## Discussion

The principal finding of this study was a significant increase in the number of broiler chicken slaughter establishments with Salmonella Enteritidis–positive broiler rinses in the years from 2000 through 2005. The 90 slaughter establishments with positive rinses were dispersed across 24 states, reflecting the geographic distribution of the US broiler industry. During the study period, increases were seen in the proportion of both large and small establishments that had such positive broiler rinses.

Some caution is warranted when interpreting our findings. The purpose of the FSIS Salmonella program is to assess performance of individual establishments. The program is not designed to estimate national prevalence of poultry contamination because it does not fully account for production volume or regional or seasonal effects. Furthermore, samples are collected after slaughter processes that are intended to reduce carcass contamination. Nonetheless, the apparent emergence of Salmonella Enteritidis in broilers is noteworthy given the increase in human Salmonella Enteritidis infection rates in the United States (*8*) and recent findings that eating chicken is a new and important risk factor for sporadic infection (*5**,**6*). Additional epidemiologic studies are recommended to further elucidate the role of contaminated chicken in human Salmonella Enteritidis infections and estimate the extent of illness attributable to chicken. Retail food surveillance and laboratory subtyping studies (*6*) may also be valuable because they enable comparisons of human and poultry strains.

In this report, 2 Salmonella Enteritidis phage types, PT 8 and PT 13, accounted for most isolates from broiler rinses. In a recent FoodNet study, the association between Salmonella Enteritidis infection and eating chicken strengthened in analyses restricted to patients infected with these 2 phage types (*6*). The possible emergence of these phage types in broiler chickens suggests that industry should implement appropriate Salmonella Enteritidis controls for broiler chickens (*17**,**18*).

The present study preceded a new FSIS policy to control Salmonella in broilers that emphasizes common serotypes of human illness (*16*). As part of this effort, FSIS held 2 public meetings on Salmonella in broilers: 1 in Athens, Georgia, in August 2005 on controls before slaughter (preharvest), and another in Atlanta, Georgia, in February 2006 on controls in the slaughter plant (postharvest). Information from these meeting was used to prepare guidelines to help broiler plants control salmonellae (*19*). The agency is also monitoring progress of meat and poultry plants in controlling this organism. If, in July 2007, most plants (e.g., 90%) that manufacture a specific product (e.g., broiler carcasses) have not reduced the percentage of Salmonella tests that are positive to at least half the FSIS performance standard, the agency will consider actions to improve control of salmonellae. One option that FSIS is considering is to post Salmonella results on the web for product classes that have not made sufficient progress, listing data by plant name.

In the 1990s, successful voluntary quality assurance programs to control Salmonella Enteritidis were developed by the egg industry and state poultry health officials (*20*). Many of the interventions are adaptable to the control of this organism in broilers. For example, control points for the organism in broilers are likely to include monitoring and sanitation of breeding flocks, hatcheries, broiler flocks, and slaughter establishments. Serotype data that FSIS provides to plants on each isolate as part of its new Salmonella policy (*16*) may also assist plant officials to make informed SE risk management decisions.

## References

[1] Patrick ME, Adcock PM, Gomez TM, Altekruse SF, Holland BH, Tauxe RV, Salmonella Enteritidis infections, United States, 1985–1999. Emerg Infect Dis. 2004;10:1–7. 15078589 10.3201/eid1001.020572 PMC3322758

[2] St Louis ME, Morse DL, Potter ME, DeMelfi TM, Guzewich JJ, Tauxe RV, The emergence of grade A eggs as a major source of Salmonella Enteritidis infections. New implications for the control of salmonellosis. JAMA. 1988;259:2103–7. 10.1001/jama.259.14.2103 3279240

[3] Trepka MJ, Archer JR, Altekruse SF, Proctor ME, Davis JP. An increase in sporadic and outbreak- associated Salmonella Enteritidis infections in Wisconsin: the role of eggs. J Infect Dis. 1999;180:1214–9. 10.1086/314984 10479150

[4] Altekruse S, Koehler J, Hickman-Brenner F, Tauxe RV, Ferris K. A comparison of Salmonella Enteritidis phage types from egg-associated outbreaks and implicated laying flocks. Epidemiol Infect. 1993;110:17–22. 10.1017/S0950268800050639 8432319 PMC2271964

[5] Kimura AC, Reddy V, Marcus R, Cieslak PR, Mohle-Boetani JC, Kassenborg HD, Chicken consumption is a newly identified risk factor for sporadic Salmonella enterica serotype Enteritidis infections in the United States: a case-control study in FoodNet sites. Clin Infect Dis. 2004;38(Suppl 3):S244–52. 10.1086/381576 15095196

[6] Marcus R, Varma JK, Medus C, Boothe EJ, Anderson BJ, Crume T, Re-assessment of risk factors for sporadic Salmonella serotype Enteritidis infections: a case-control study in five FoodNet sites, 2002–2003. Epidemiol Infect. 2006;7:1–9. 16756692 10.1017/S0950268806006558 PMC2870546

[7] Cowden JM, Lynch D, Joseph CA, O'Mahony M, Mawer SL, Rowe B, Case-control study of infections with Salmonella Enteritidis phage type 4 in England. BMJ. 1989;299:771–3. 10.1136/bmj.299.6702.771 2508916 PMC1837639

[8] Centers for Disease Control and Prevention. Preliminary FoodNet data on the incidence of infection with pathogens transmitted commonly through food—10 states, United States, 2005. MMWR Morb Mortal Wkly Rep. 2006;55:392–5. 16617286

[9] Food Safety and Inspection Service. Pathogen reduction; hazard analysis and critical control point (HACCP) systems; final rule. Fed Regist. 1996;61:38805–55 [cited 2006 Oct 11]. Available from http://www.fsis.usda.gov/OPPDE/rdad/FRPubs/93-016F.htm

[10] Food Safety and Inspection Service. FSIS directive 10,230.5: Self-instruction guide for collecting raw meat and poultry product samples for Salmonella analysis. February 4, 1998. [cited 2006 Oct 11]. Available from http://www.fsis.usda.gov/OPPDE/rdad/FSISDirectives/10230-5.pdf

[11] Food Safety and Inspection Service. Microbiology laboratory guidebook. August 17, 2006 [cited 2006 Oct 11]. Available from http://www.fsis.usda.gov/Science/Microbiological_Lab_Guidebook

[12] Ewing E, ed. Edwards and Ewing's identification of Enterobacteriaceae. 5th ed. London: Elsevier; 1986.

[13] Ward LR, de Sa JD, Rowe B. A phage-typing scheme for Salmonella Enteritidis. Epidemiol Infect. 1987;99:291–4. 10.1017/S0950268800067765 3315705 PMC2249269

[14] Laconcha I, Baggesen DL, Rementeria A, Garaizar J. Genotypic characterisation by PFGE of Salmonella enterica serotype Enteritidis phage types 1, 4, 6, and 8 isolated from animal and human sources in three European countries. Vet Microbiol. 2000;75:155–65. 10.1016/S0378-1135(00)00208-X 10889406

[15] USDA National Agricultural Statistics Service. Number of broilers and other meat-type chickens sold: 2002 [cited 2006 Oct 11]. Available from http://www.nass.usda.gov/research/atlas02/Livestock/Poultry/Number%20of%20Broilers%20and%20Other%20Meat-Type%20Chickens%20Sold.gif

[16] Food Safety and Inspection Service. Salmonella verification sample result reporting: agency policy and use in public health protection. Fed Regist. 2006;71:9772–7.

[17] Gast RK, Holt PS. Experimental horizontal transmission of Salmonella Enteritidis strains (phage types 4, 8, and 13a) in chicks. Avian Dis. 1999;43:774–8. 10.2307/1592747 10611994

[18] McIlroy SG, McCracken RM, Neill SD, O'Brien JJ. Control, prevention and eradication of Salmonella Enteritidis infection in broiler and broiler breeder flocks. Vet Rec. 1989;125:545–8. 10.1136/vr.125.22.545 2690452

[19] Food Safety and Inspection Service. Compliance guideline for controlling Salmonella in poultry. 1st ed. August 2006 [cited 2006 Oct 11]. Available from http://www.fsis.usda.gov/PDF/Compliance_Guideline_Controlling_Salmonella_Poultry.pdf#search=%22Compliance%20guideline%20for%20controlling%20Salmonella%20in%20poultry%22

[20] Mumma GA, Griffin PM, Meltzer MI, Braden CR, Tauxe RV. Egg quality assurance programs and egg-associated Salmonella Enteritidis infections, United States. Emerg Infect Dis. 2004;10:1782–9. 15504264 10.3201/eid1010.040189 PMC3323249

